# The roles of Hippo/YAP signaling pathway in physical therapy

**DOI:** 10.1038/s41420-024-01972-x

**Published:** 2024-04-26

**Authors:** Chunran Pan, Xiaoxia Hao, Xiaofeng Deng, Fan Lu, Jiawei Liu, Wenjie Hou, Tao Xu

**Affiliations:** 1 Department of Rehabilitation, Tongji Hospital, Tongji Medical College, Huazhong University of Science and Technology, Wuhan, China; 2 Department of Orthopedics, Tongji Hospital, Tongji Medical College, Huazhong University of Science and Technology, Wuhan, China

**Keywords:** Diseases, Translational research

## Abstract

Cellular behavior is regulated by mechanical signals within the cellular microenvironment. Additionally, changes of temperature, blood flow, and muscle contraction also affect cellular state and the development of diseases. In clinical practice, physical therapy techniques such as ultrasound, vibration, exercise, cold therapy, and hyperthermia are commonly employed to alleviate pain and treat diseases. However, the molecular mechanism about how these physiotherapy methods stimulate local tissues and control gene expression remains unknow. Fortunately, the discovery of YAP filled this gap, which has been reported has the ability to sense and convert a wide variety of mechanical signals into cell-specific programs for transcription, thereby offering a fresh perspective on the mechanisms by which physiotherapy treat different diseases. This review examines the involvement of Hippo/YAP signaling pathway in various diseases and its role in different physical therapy approaches on diseases. Furthermore, we explore the potential therapeutic implications of the Hippo/YAP signaling pathway and address the limitations and controversies surrounding its application in physiotherapy.

## Facts

- The Hippo/YAP signaling pathway plays a crucial role in various disease.
- Hippo/YAP signaling pathway is involved in the process of regulating mechanotransduction.
- Physiotherapy induces mechanical stimulation or environmental changes in local tissue.

## Open questions

- What diseases that regulated by Hippo/YAP signaling pathway through regulating mechanotransduction?
- What are the links between Hippo/YAP signaling pathway and physiotherapy?

## Introduction

In recent years, extensive research has been reported the association between Yes-associated protein (YAP) and various diseases owing to its diverse functions. Particularly, Hippo/YAP signaling pathway plays a crucial role in the regulation of tissues and organs development, homeostasis, and regeneration [1]. The influence of mechanical stimulation on cell behavior is complex, as it can impact the cell fate by altering cell mechanics and shape [2]. Mechanical forces are the main regulators of YAP/TAZ in multicellular contexts, and it operates independently of large tumor suppressor (LATS) [3, 4]. As both a sensor and mediator of diverse mechanical signals, Hippo/YAP signaling pathway regulate multiple cellular behaviors and processes, including cell proliferation, differentiation, and migration through interacting with other molecules and signaling pathways.

Notably, physical therapy is a large class related to evidence-based therapeutic, which has been extensively utilized for the treatment of physical disorders caused by trauma or other medical conditions such as musculoskeletal, cardiovascular, and neurological origins. Cold therapy, heat therapy, ultrasound, vibration, and exercise are main treatment approach techniques. Despite the validated clinical effectiveness of these therapies, their specific mechanisms of action remain incompletely understood [5, 6]. It is worth noting that many of these therapies induce mechanical stimulation or environmental changes in local tissue cells. Consequently, investigating how cells perceive and transduce these mechanical stimuli into chemical signals is crucial for comprehending the therapeutic effects of physiotherapy. Therefore, the objective of this review aims to provide a summary of the role of Hippo/YAP signaling pathway in the context of physical therapy for disease treatment and explore its potential significance in disease management.

## The Hippo/YAP signaling pathway

Hippo/YAP signaling pathway is a highly conserved signaling pathway responsible for regulating organ size and tissue growth, which is initially discovered in Drosophila [7]. Subsequently, some studies demonstrated that YAP also controls cell proliferation and differentiation in mammals [8]. The components of this pathway include mammalian Ste20-like kinase 1 (MST1; also known as STK4), MST2 (also known as STK3), Salvador 1 (SAV1) (Sav in D. melanogaster) adaptor proteins, LATS1, LATS2 (Warts in D. melanogaster), MOB kinase activator 1A (MOB1A) and 1B (MOB1B) proteins (Mats in D. melanogaster), transcriptional co-activators YAP and transcriptional co-activator with PDZ-binding motif (TAZ) (Yorkie in D. melanogaster), and TEAD transcription factors (TEAD1-TEAD4) (Scalloped in D. melanogaster) [9].

**Fig. 1:**
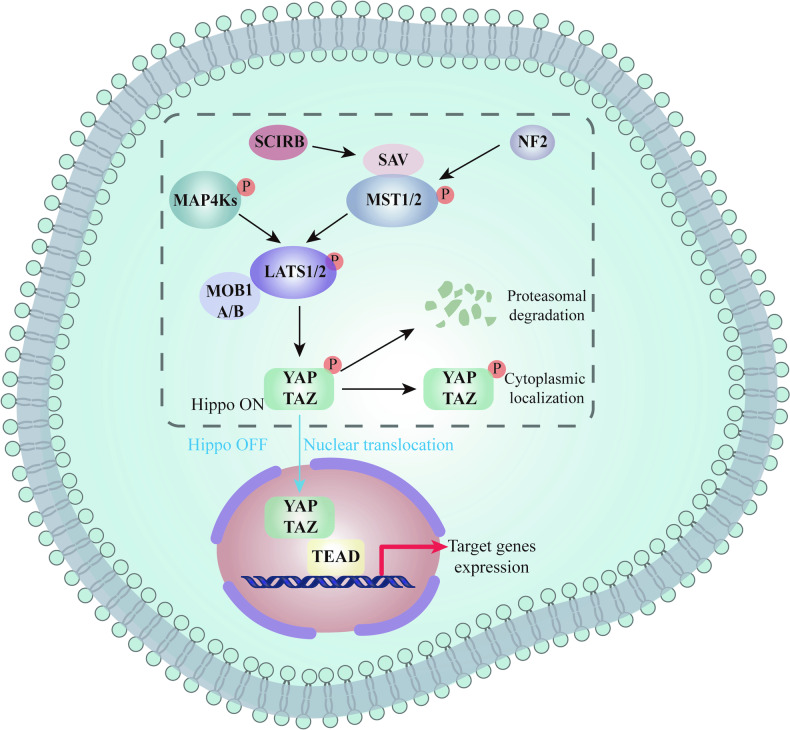
The model of Hippo sinaling pathway in mammalian cells and the activity of Hippo sinaling pathway controls the dynamic localization of YAP/TAZ between nucleus and cytoplasm. When Hippo sinaling pathway is turned off, YAP/TAZ is dephosphorylated and accumulates in the nucleus, which subsequently binds to TEADs to induce gene transcription. When the Hippo pathway is on, active LATS kinases phosphorylate YAP/TAZ, leading to cytosolic retention and degradation.

According to reports, several upstream proteins have been identified to regulate the activity of Hippo/YAP signaling pathway kinases. These proteins include members of the Ras-association domain-containing family, neurofibromin 2, Ajuba, angiomotin, kidney, brain protein, and zonula occludens. Additionally, the transcriptional co-activators YAP and TAZ can interact with TEAD family transcription factors, such as SOX9, connective tissue growth factor, and cysteine-rich protein 61 [10]. Activating Hippo signaling pathway triggers a series of phosphorylation events mediated by MST and LATS kinases, which leads to the phosphorylation of YAP/TAZ, causing their retention in the cytoplasm (Fig. 1). Consequently, they undergo degradation by proteasome, leading to the inhibition of their transcriptional activity. Conversely, when the Hippo signaling pathway is deactivated, YAP/TAZ translocates to the nucleus and interacts with TEAD or other transcription factors, which regulates downstream signaling molecules associated with cell proliferation, apoptosis, differentiation, and maturation. However, it is worth noting that the YAP-TEAD complex alone is incapable of fully activating biological program in vivo. Hence, YAP necessitates interaction with various transcription factors, thereby governing diverse cellular signaling pathways, and executing multiple biological functions [11–14].

Additionally, Hippo/YAP signaling pathway also exhibits interaction with DNA binding transcription factors such as SMAD1, SMAD7, RUNX, and transcription factor TBX5 [15–18], resulting in its ability in regulating tumor growth and tissue regeneration [19]. Furthermore, YAP also monitors alterations in mechanical forces, extracellular matrix (ECM) stiffness, cytokines, growth factors, and hypoxia, thereby acting as the primary sensor for cell structure, morphology, differentiation, and proliferation.

## Hippo/YAP is involved in the diseases procession as a mechanical transducer

### Hippo/YAP and cancer

It has been reported that in various malignant tumors, mechanical signals played a significant role in the development of cancer cells by regulating Hippo/YAP signaling pathway [20–22]. For example, ECM stiffness is one of the risk factors for breast cancer, which promotes the proliferation and invasive phenotype of breast epithelial cells [23, 24]. Another study also demonstrated that mechanical signals played a key role in human breast cancer cells. As a mechanoresponsive oncogenic Hippo signaling effector, YAP was positively associated with breast cancer and directly promoted Skp2 transcription. While YAP inactivation induced cell cycle exit through downregulating of Skp2, thereby inhibiting YAP-induced tumororigenesis [25]. Fan et al. found that advanced glycation end-products (AGEs) promoted changes in collagen structure and improved ECM viscoelasticity. High AGEs promoted the induction of hepatocellular carcinoma through binding to oncogenic β-catenin signaling, whereas inhibiting the production of AGEs inhibited hepatocellular carcinoma growth. Mechanistically, animal and cell studies indicated that increased viscoelasticity promoted proliferation and invasion of hepatocellular carcinoma cells via integrin-β1-tensin-1-YAP mechanotransductive pathway [26]. These studies indicate that YAP can sense and transduce mechanical signals changes, thereby affecting cell survival and tumor growth by regulating its phosphorylation and nuclear translocation.

### Hippo/YAP and regeneration

Due to the special role of YAP in signal transduction, Hippo/YAP signaling pathway assumes a crucial function in the the regeneration of tissues and organs [27–29]. As reported, liver had good regenerative capacity after injury, which was related to mechanical changes caused by flow [30, 31]. Li et al. found that after stimulated by mechanical stress, primary mouse hepatocytes were able to re-enter the cell cycle in a YAP-dependent manner, and initiated hepatocyte proliferation. Activation of β1 integrin significantly increased shear-initiated hepatocyte proliferation when mechanical signals were extracellular, suggesting the critical role of β1 integrin as a shear sensor on the cell membrane. When mechanical signals were present in the nucleus, activated YAP was able to transport into the nucleus and trigger the transcription of various effector molecules [32]. Additionally, another study reported that mechanical stress promoted the activation of Engrailed-1 through classical mechanotransduction signaling. However, blocking mechanical transduction signaling with the YAP inhibitor verteporfin or transgenic YAP knockdown prevented Engrailed-1 activation, leading to wound regeneration of Engrailed-1 lineage-negative fibroblasts and restoration of skin attachment and mechanical strength [33].

### Hippo/YAP and musculoskeletal diseases

Muscles, bones, and cartilage are the main weight-bearing and activity generation tissues of body, there are sensitive to mechanical signals and regulated by Hippo/YAP signaling pathway [34]. For example, mechanical stress has been reported to regulate cell cycle progression of chondrocytes through YAP. Mechanically, YAP activation increased the expression of cell cycle-related proteins PCNA and cyclin D1, thereby enhancing cell cycle progression, while inhibition of YAP inhibited cell cycle-promoting effect of mechanical stress [35]. In addition, mechanical stress also regulates the chondrocyte phenotype by affecting the nuclear translocation of YAP. When extracellular mechanical stress increased significantly, the expression of YAP raised, leading to dedifferentiation of chondrocytes and loss of chondrocyte properties. However, after cytoskeleton disruption using CytoD, YAP cytoplasmic retention was increased, resulting in the decrease of chondrocytes’ ability to respond to mechanical stress, and their phenotype was maintained [36]. Similarly, another study showed that the expression of nuclear YAP was lower and Col2a1 expression was significantly higher in soft substrate than in stiff substrate, whereas YAP knockdown restored the low level of collagen II expression caused by stiff substrate, indicating that YAP maintain the cartilage phenotype in response to mechanical stress [37].

Notably, it has been reported that pathological activation of the Hippo/YAP signaling pathway was associated with osteogenic differentiation. For example, Zhong et al. verified that Piezo1 activated by mechanical stress was able to regulate glutaminase 1 (GLS1)-mediated glutaminolysis and advance osteogenic differentiation of valve interstitial cells, whereas inhibition or knockdown of Piezo1 and GLS1 respectively diminished these effects. Mechanistically, activation of Piezo1 promoted calcium-dependent YAP activation, thereby regulating GLS1-mediated glutaminolysis and enhancing osteogenic differentiation through histone acetylation of runt-related transcription factor 2 promoter [38]. Another study by Li suggested that mechanical stress accelerated the osteogenesis and angiogenesis of tissue-engineered laminae through the F-actin/YAP-1/β-Catenin signaling axis. Mechanistically, F-actin sensed the surrounding mechanical signals and balanced the tensional state through reorganization, leading to nuclear translocation of YAP/TAZ and downstream transcriptional activities, while mechanical stress increased the expression and nuclear translocation of β-Catenin and facilitated the binding of YAP1 and β-Catenin [39].

Nevertheless, in addition to above diseases, YAP has also been reported to participate in the regulation of the pathological progression of diseases, including skin aplasia and cardiomyocyte hypertrophy by responding to various mechanical signals inside or outside the cells [40, 41]. Therefore, it is necessary to summarize the studies related to the mechanism by which YAP convers mechanical stimuli into chemical signals and affects cell fate, thereby providing insights into treating disease by altering cellular mechanical dynamics.

## Mechanical stress affect disease progression by regulating YAP

### Mechanical stress affect cancer progression by regulating YAP

It is well known that in solid tumors, mechanical forces in the tumor microenvironment undergo significant changes due to alterations in local fluid motion and increases in interstitial pressure, thereby affecting the survival of cancer cell [42, 43]. As reported, YAP is a protein that responds to physical stimuli such as cell shape, ECM elasticity and cell density. Therefore, manipulating mechanical signals potentially accelerates the cancer progression by regulating the expression and activity of YAP [42, 44]. One example of this is the activation of YAP induced by fluid wall shear stress (WSS), which has been found to increase invasiveness of cancer cells. Furthermore, WSS has also been shown to activate Piezo1 and trigger signaling cascades within the cytoplasm through biomechanical stimulation, leading to increased nuclear localization of YAP/TAZ, ultimately affecting prostate cancer progression [45]. Interestingly, YAP is an important molecule that senses shear stress induced by blood or lymph flow, thereby promoting the survival and migration of clear cell renal cells in circulation. Mechanically, low shear stress increased the activation and nuclear localization of YAP, while YAP knockdown directly interrupted epithelial-mesenchymal transition (EMT) induced by low shear stress. Moreover, YAP inhibition increased high shear stress-induced cell apoptosis, suggesting that YAP might promote tumor metastasis by inducing EMT and protect tumor cells from anoikis and cell apoptosis induced by high shear stress in vasculature [46]. Another study by Zhao showed that suspension state and shear stress improved the EMT of breast tumor cells (BTCs). Mechanistically, shear stress in suspension state promoted the EMT of cells. When cells were under shear stress in suspension state, the function of YAP in promoting transcription was decreased, thereby reducing the expression of targeted microRNA-29b. Therefore, YAP promoted cell EMT and thereby enhanced cell migration under the combination of suspension state and shear stress [47].

### Mechanical stress governs vascular homeostasis by regulating YAP

**Fig. 2:**
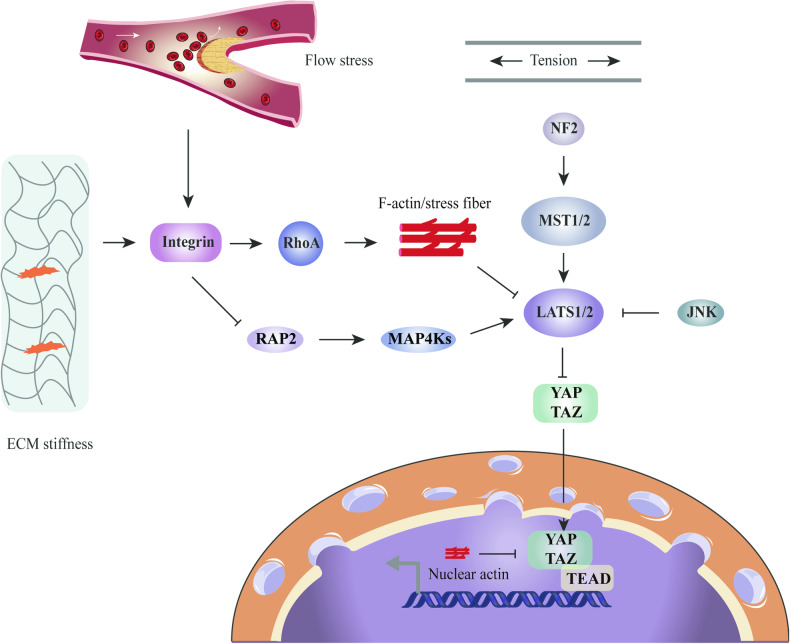
Schematic representation of the molecular mechanism by which mechanical cues regulate YAP/TAZ. Mechanical cues, including ECM stiffness, externally applied mechanical stretch, and flow stress, control YAP/TAZ activity through Hippo dependent or independent pathways. the core kinase cascade consisting of MST1/2 and LATS1/2 responds to mechanical cues that regulate YAP/TAZ phosphorylation and localization. Mechanical cues also bypass these kinases and act through cytoplasmic and nuclear actin to regulate YAP/TAZ localization.

In endothelial cells (ECs), Hippo/YAP signaling pathway also plays an important role in determining the fate of cells in response to shear stress [48]. As reported, owing to ECs were continuously exposed to mechanical forces generated by blood flow, shear stress played an important role in governing vascular homeostasis, with different blood flow patterns exerting diverse stresses on the vessel wall, thereby influencing the phenotypes of ECs and determining the occurrence and distribution of atherosclerotic lesions [49]. For example, stable unidirectional flow exhibited its protective ability to safeguard the endothelium through acting as an antioxidant, anti-inflammatory, and anti-atherosclerotic agent. Conversely, disturbed flow showed detrimental effects by stimulating oxidative stress, inflammation, and atherogenesis [50]. Atherosclerosis is a mechanobiology-related disease that occurs preferentially in the aortic arch and arterial branches, which are exposed to disturbed flow [51]. Xu et al. suggested that laminar flow reduced YAP nuclear translocation in human ECs. Moreover, they found that the activity of YAP induced by laminar flow and disturbed flow was different in ECs, which might differentiate the type of blood flow and thus have different effects on the vessel wall [52]. Therefore, inhibition YAP activity in response to laminar flow could maintain vascular endothelial homeostasis and prevent the occurrence of atherosclerosis. Another study also suggested that unidirectional shear stress delayed atherosclerosis by inhibiting YAP activity. Mechanistically, unidirectional shear stress increased RhoA inhibition and YAP inhibition via activating integrins and promoting integrin-Gα_13_ interactions. Subsequently, YAP/TAZ inhibition suppressed JNK signaling and down-regulated proinflammatory gene expression, thereby decreasing monocyte attachment and infiltration. In addition, in vivo, CRISPR/Cas9-mediated YAP knockdown in endothelium also reduced plaque formation in ApoE mice [53]. Additionally, Yuan et al. discovered that laminar flow inhibited the Hippo/YAP pathway though autophagy in vascular ECs, thereby preventing atherosclerosis and blocking the formation of atherosclerotic plaques. Mechanically, through regulating endothelial autophagy, laminar flow promoted YAP degradation and inhibited the Hippo pathway in vascular ECs and increased the expression of SIRT1, which mediated YAP deacetylation and promoted nuclear YAP export and degradation through autophagy [54]. Furthermore, a study by Li found that oscillatory shear stress (OSS) induced tyrosine phosphorylation and sustained nuclear translocation of YAP in ECs to activate endothelial atherogenic, which was dependent on integrin α5β1 activation. Whereas YAP overexpression in ECs attenuated the anti-atheroprone effect of an integrin α5β1-blocking peptide (ATN161) in Apoe-/-mice. Mechanically, activation of integrin α5β1 and its downstream kinase c-Abl mediated OSS-induced YAP nuclear translocation. Therefore, c-Abl inhibition reduced the OSS-induced EC activation and the development of early-stage atherosclerosis [55]. Taken together, regulating the activity of YAP by altering the mechanical stimuli on tissues and cells may affect the occurrence and development of diseases (Fig. 2).

## The impact of physical therapy on disease via Hippo/YAP signaling pathway

### Exercise and YAP

Exercise training is frequently recommended for the prevention and treatment of diseases [56]. The mechanical stimulation generated by exercise training may improve bone quality and cardiopulmonary function, leading to new strategies for the therapeutics of chronic diseases including osteoporosis and cardiopulmonary dysfunction [57]. For example, under dynamic culture conditions, primary cells from patients with heterotopic ossification showed significantly raised expression of osteogenic marker genes compared to static culture, indicating that mechanical stimulation may promote osteogenic differentiation of ligaments [58]. Zhu et al. found that mechanical stimulation had a significant effect on osteoblast differentiation at cellular level, which was related to the nuclear translocation of the mechanical signaling response factor YAP [59]. On this basis, they used CRISPR/Cas9 technology to establish EIIa^cre^-Enpp1^flox/flox^ gene-deficient mice and demonstrated that mechanical stimulation played a key role in the pathological process of heterotopic ossification of tendons and ligaments. They also verified that YAP expression was significantly increased at the sites of ligament heterotopic ossification. In addition, the ossification of specific segments was significantly enlarged in the treadmill trained mice compared to controls [60]. Moreover, Tao et al. found that Hippo/YAP signaling pathway might mediate doxorubicin-induced cardiotoxicity by regulating vascular injury and cardiomyocyte apoptosis [61]. Interestingly, exercise training during or after doxorubicin treatment demonstrated a significant reduction in both early and late cardiotoxicity. At cellular level, exercise reversed doxorubicin-induced reductions in cardiovascular pericytes and ECs, abnormal mitochondria, vacuolization, and increased autophagosomes [62]. Notably, it has been reported that YAP promoted cellular mobilization for cardiac regeneration and repair, stimulated cardiomyocyte proliferation, and accelerated cardiac regeneration after myocardial infarction [63]. By suppressing Hippo/YAP signaling pathway, exercise training inhibited cardiomyocyte apoptosis and enhanced the migration of bone marrow stem cells to the heart and their differentiation into ECs and pericytes, thereby ameliorating doxorubicin-induced decrease in cardiac function [61]. In contrast, specifically targeted knockout of YAP in mouse cardiomyocytes resulted in increased cardiomyocyte apoptosis and fibrosis, as well as decreased cardiac function after acute myocardial infarction [64]. Xi et al. observed that aerobic training played a crucial role in inhibiting the cardiac pathological remodeling and safeguarding the myocardium in rats with myocardial infarction. Mechanically, by promoting proliferation of H9C2 cells, aerobic training kept more cardiomyocytes alive and increased angiogenesis through regulating YAP phosphorylation and nuclear translocation to activate the APJ-Akt signaling pathway [65].

### LIPUS and YAP

Mechanical signals are important for organ development and homeostasis, which regulate cellular functions such as migration, proliferation, differentiation, and apoptosis [66]. Over the past decade, ultrasound, specifically low-intensity pulsed ultrasound (LIPUS), has been demonstrated to enhance tissue regeneration in a safe and non-invasive manner [67, 68]. Notably, LIPUS is widely used in clinical applications and basic research due to its minimal thermal effects and intensity. The output frequencies of LIPUS typically range from 1 to 3 MHz, with a treatment intensity falling within the range of 0.02–1 W/cm^2^ spatial average temporal average [69–73]. Therefore, periodic sound waves generated by ultrasound can induce vibration and collision in target tissues, thereby modifying the local microenvironment of cells [74].

As reported, YAP responds to mechanical stimuli by regulating the transcription profile of actin fibers, thereby promoting tissue regeneration through the control of cell proliferative potential [75, 76]. For example, mechanical stimulation induced by focused low-intensity pulsed ultrasound (FLIPUS) stabilized the actin cytoskeleton, thereby reducing phosphorylation of YAP at Ser127 and increasing the activity of YAP. Subsequently, through regulating the expression of proliferation genes, including amphiregulin (AREG), cysteine-rich angiogenic inducer 61(Cyr61), cyclinD1, and the cell division-related gene anillin (ANLN), FLIPUS promoted cell proliferative potential and tissue regeneration process [77]. Additionally, overexpression of YAP has been shown to enhance the proliferation and migration of vascular smooth muscle cells [78]. By promoting the phosphorylation and nuclear translocation of YAP, LIPUS strengthened endothelial cell connections, thereby facilitating vascular remodeling, initiating angiogenesis, and expediting wound healing. Mechanically, they found that in human umbilical vein ECs, phosphorylation YAP was more significantly at 0.5 h than at 2, 6, and 12 h after LIPUS treatment. Meanwhile, the nuclear translocation of the YAP was most obvious at 0.5 h after treatment, while siYAP reduced YAP nuclear translocation, and shLATS1/2 increased YAP nucleus translocation [79]. These studies indicating that mechanical stimulation produced by LIPUS promote the regeneration process of tissues, providing a theoretical basis for the application of LIPUS in the treatment of diseases such as fracture.

Moreover, LIPUS suppressed the differentiation of adipocytes, making LIPUS a new therapeutic strategy for suppressing obesity. When applied to LIPUS, adipocytes displayed actin stress fibers and more nucleus-localized YAP. Besides, the production of CCN2 was also enhanced, which is the target gene of YAP. However, adipocytes without LIPUS treatment showed cell rounding and cortical actin structure. Mechanically, the actin cytoskeleton formed actin stress fibers and reduced G-actin when LIPUS applied to adipocytes. Subsequently, the actin cytoskeleton dynamics promoted YAP retention in the nucleus, leading to the induction of CCN2, which reduced adipocyte differentiation by suppressing PPARγ gene expression [80, 81]. In addition, LIPUS inhibited apoptosis of retinal ganglion cells induced by optic nerve crush in a YAP-dependent manner. Mechanically, LIPUS improved YAP activation, nuclear translocation, and p-YAP inhibition in vivo and in vitro, thereby safeguarding retinal ganglion cells against mitochondrial damage and triggering retinal protection via cleaving caspase-3 and activating cyclin E1. Nevertheless, inhibition of YAP abolished the anti-apoptotic effect induce by LIPUS [82], which provides novel insight for LIPUS protection on retinal ganglion cells. In addition to participate in cell proliferation, differentiation and apoptosis, YAP also affects cell survival by regulating autophagy in response to inflammatory microenvironment [83]. A study by Jian et al. demonstrated that LIPUS alleviated the apoptosis of periodontal ligament cells by upregulating the expression and nuclear translocation of YAP and promoting autophagy completion. On the contrary, the reduction of YAP expression enhanced apoptosis and the completion of autophagy in LPS-treated periodontal ligament cells [84]. Another study also suggested that LIPUS could delay the progression of osteoarthritis by restoring autophagy in chondrocytes via YAP-RIPK1-NF-κB axis. By inhibiting the phosphorylation of YAP and the binding of YAP to RIPK1, LIPUS significantly downregulated the expression of inflammation-related molecules and rescued the impaired autophagy in chondrocytes. In vivo, LIPUS also significantly restored cartilage damage and subchondral bone loss in OA rats [85]. These studies suggest that the mechanical stimulation produced by LIPUS regulate the phosphorylation and nuclear translocation of YAP, thereby regulating cell survival and achieving the purpose of disease treatment.

### Temperature and YAP

Temperature has been recognized as a crucial determinant of disease outcome [86]. Furthermore, hyperthermia or cold therapy is commonly employed to alleviate pain, reduce swelling, and enhance blood circulation in clinical settings [87]. It has been reported that YAP participated in the regulatory processes of temperature and initiated the transcriptome associated with heat shock [88]. For instance, YAP plays a significant role in the response to heat stress, as it was activated through dephosphorylation during heat shock, leading to enhanced heat shock transcriptome and cell survival [89, 90]. Bone defects, especially large bone defects, are hard to recover and may lead to nonunion [91]. Shi et al. found that heat stress triggered the migration of YAP into the nucleus through LATS1 dephosphorylation and degradation. This nuclear migration of YAP contributed to enhanced expression of its target gene TG2, which was necessary for the activation of heat stress factors and consequently regulated the differentiation of ectomesenchymal stem cells towards osteogenesis [92].

Furthermore, YAP has been identified as a potential protein responsible for cold shock during brain development. Under cold stress, the expression of YAP was observed to increase in the developing cerebral cortex. During embryonic stages, RBM3 maintained the stable expression of YAP by binding to the 3’UTR domain of YAP mRNA, which governed the proliferation and differentiation of neural stem cells [93]. Additionally, YAP plays an important role in the thermogenic activity of brown adipose tissue (BAT) and collaborates with TEAD to regulate uncoupling protein 1 (UCP1) transcription [94]. When exposed to low temperatures, YAP expression was found to be higher in BAT compared to inguinal white adipose tissue (iWAT). The regulation of YAP expression was mediated by miR-429, overexpression of miR-429 reduced the induction of UCP1 induced by cold exposure. Interestingly, overexpression of YAP in middle-aged mice alleviated the impact of cold exposure-induced UCP1 expression and browning of WAT. However, in young mice, YAP overexpression solely raised basal UCP1 levels in iWAT [95].

Intriguingly, specific low-temperature materials have demonstrated their ability to treat disease by regulating YAP activation. For instance, Kim et al. found that the activation of YAP was regulated by low-temperature argon plasma (LTAP) in a melanocortin 1 receptor-dependent manner, leading to an increase in the expression of genes associated with skin barrier and moisturizing factors. Mechanistically, LTAP promoted the activation of YAP, thereby regulating the expression of factors related to melanogenesis, suppressing the growth of melanoma cells and modulating the activity against melanin production. On the contrary, knockdown of YAP or use the inhibitor of YAP leading to the downregulation of the expression of moisturizing-related and melanogenesis-related factors [96]. Additionally, biomaterials engineered with specific bioactive ligands and tunable mechanical properties are also vital in the process of tissue repair [97]. By means of intercellular adhesion, cells were capable of transmitting enduring internal as well as external mechanical forces [98, 99], thereby influencing the activation of biochemical signaling pathways and downstream gene transcription [100]. For example, mechanical stretch has been shown to promote the expansion of intact skin by influencing gene regulation of skin stem cells [101]. Li et al. identified that the healing of wounds through contraction relied on important mechanical stimulation sensors such as YAP. By exploiting the mechanical sensitivity mediated by YAP, temperature-sensitive hydrogel enhanced the proliferative activity of basal cells, leading to a reduction in inflammation and an improvement in wound healing [102]. These studies suggest that physical therapy can promote wound healing by regulating the temperature and mechanical stress in the surrounding environment of tissue, providing new insights into the therapeutics of dermatological and metabolic diseases.

### Vibration and YAP

Low-intensity vibration (LIV) is a mechanical stimulation with acceleration ranging from 0.1 to 2 g and frequency between 20 and 200 Hz. These vibrations have been found to have anabolic and/or anticatabolic effects on tissues. Studies have demonstrated that vibrations enhanced trabecular bone density and volume, improved bone hardness and strength, and slowed down bone loss caused by disuse [103–106]. Additionally, LIV also increased muscle contractility, strength, and cross-sectional area [107–109]. Under mechanical stimulation, mesenchymal stem cells (MSCs) play an important role in preserving and repairing bone, which is beneficial for astronauts, injured personnel on prolonged bed rest, and inactive older adults. The application of LIV has been shown to increase MSC contraction, activate RhoA signaling, and enhance increased osteogenic differentiation and MSC proliferation, thereby improving bone and muscle indicators at tissue level [110–112]. In addition, YAP and TAZ are important regulators of the function and expression of Runx-2, which is the major osteogenic transcription factor in stem cells [113]. Consequently, the loss of YAP and TAZ result in skeletal defects [114]. Touchstone et al. indicated that under simulated microgravity (SMG) conditions, daily application of LIV increased cellular YAP, while loss of nuclear structural elements played a role in cell proliferation reduction by altering YAP function [115]. Another study found that LIV restored nuclear YAP levels and acute YAP nuclear transport in SMG-treated MSCs. Mechanically, SMG exacerbated the damage of acute YAP nuclear entry while acute LIV and LPA treatments promoted nuclear YAP entry in SMG-treated MSCs. However, daily administration of LIV reinstated the SMG-driven decline in basal nuclear YAP to control levels as well as enhanced the LPA-induced but not acute LIV-induced YAP nuclear entry [116]. These studies suggest that mechanical stress affect the growth and differentiation of MSC by regulating YAP nuclear localization, thereby promoting bone healing after fracture and reducing musculoskeletal degeneration and bone loss in the elderly, injured personnel, and astronauts in microgravity.

Low-intensity extracorporeal shock wave therapy (LIESWT) is a mechanical stimulation method that utilizes special probes to deliver vibration energy to local tissues to treat diseases [117, 118]. It has been extensively studied in various diseases, including coronary artery disease, musculoskeletal injury, neurodegenerative disease, and erectile dysfunction [119]. During the administration of LIESWT, targeted tissue experience high-frequency squeezing and shear forces, primarily at the interface of diverse media (with diverse intrinsic properties of wave reflection and refraction), like periosteum, peritoneum, or perineurium locations. As reported, mechanical stress played an important role in the development and regeneration of peripheral nerve [119]. Schwann cell is a specialized cell in the peripheral nerve tissue, which perceives mechanical cues through specific molecules in the ECM, such as laminin, collagen, and integrins. Notably, YAP has been identified as a key regulator of Schwann cell proliferation, differentiation, and myelination [120], which regulating the peripheral myelination and the expression of laminin receptors of Schwann cells [121, 122]. A study by Li et al demonstrated that LIESWT promoted nerve regeneration and functional recovery after sciatic nerve injury. Mechanistically, LIESWT upregulated the expression level and nuclear translocation of YAP, which acted as an integrated transcriptional complex for related gene expression modification, thereby promoting the activation of rat Schwann cells. Nevertheless, the activation process of Schwann cells was significantly inhibited in the circumstances of TAZ knockdown in vitro [123]. Histological and functional recovery after peripheral nerve injury has always been a challenge due to the long distances of surgical repair from the innervating terminal organs, and the above researches provide a direction for physical therapy on promoting the recovery of injured nerves.

### Electroacupuncture and YAP

Electroacupuncture (EA) is an acupuncture treatment that combines modern electrical stimulation with traditional acupuncture [124]. As a unique treatment method, EA is safe, effective, and has few side effects, which has been used to treat a variety of chronic diseases [125]. Ischemic stroke is a disease with high mortality and high disability rate. In recent years, a large number of studies have proved that EA improved the neurological function of patients with ischemic stroke and promoted the recovery of motor function in the later stage [126, 127]. As reported, the expression of YAP increased in the cerebral cortex of rats after cerebral ischemia-reperfusion injury [128]. Through regulating suppressor of cell signaling 3, YAP negatively regulated inflammatory pathways and aggravated neuroinflammatory responses [129]. Furthermore, the interaction between Connexin 43 and YAP promoted nuclear translocation of YAP and regulated the activation of astrocytes, thereby participating in the process of ischemic brain injury in mice with intracerebral hemorrhage [130]. An animal study found that EA upregulated the expression of YAP in the penumbra area of brain, thereby reducing apoptosis and neuroinflammation, and improving cerebral ischemia reperfusion injury in rats [131]. Besides, Liang et al. demonstrated that EA activated the expression of YAP, OPA1, MFN2 and MFN1, downregulated the expression level of pro-apoptotic factor BAX, and activated the mitochondrial fusion function, thereby reducing cerebral cortical injury and apoptosis in rats with middle cerebral artery focal cerebral ischemia/reperfusion. However, blocking YAP with verteporfin inhibited activation of YAP by EA and acerated cerebral ischemia/reperfusion injury in rats [132]. Another animal study also found that EA downregulated Lnc826 in rats with middle cerebral artery occlusion, thereby reducing microglial activation and inflammation, and regulating microglial polarization through the Lnc826-mediated Hippo/YAP signaling pathway, thereby promoting ischemic brain damage [133].

## Therapeutic potential and current constraints

To date, with the continuous advancement of rehabilitation medicine and deeper understanding of the concept of rehabilitation, physical therapy is increasingly employed to alleviate chronic pain and address various chronic illnesses. However, the specific mechanism underlying its therapeutic effects remain unclear. Fortunately, Hippo/YAP signaling pathway fills this knowledge gap due to its mechanotransduction properties. Over the past decade, increasing evidences have indicated the involvement of Hippo/YAP signaling pathway in disease progression regulation through physical therapy. Consequently, it is imperative to focus on this pathway to investigate the mechanism of mechanical signal transduction in physical therapy, which can establish a foundation for the development of novel treatment programs in clinical practice.

**Fig. 3:**
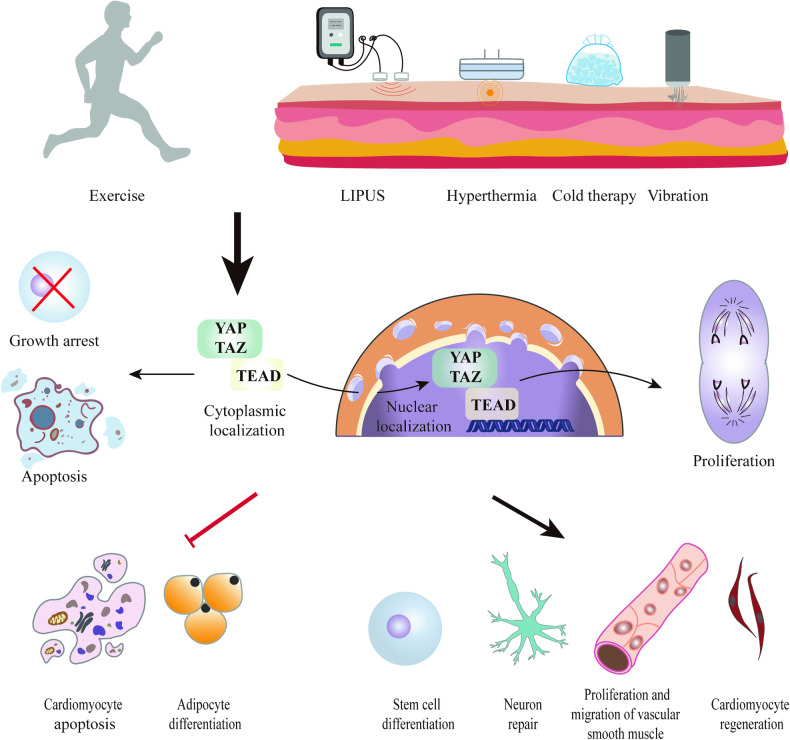
Changes of mechanical stimulation and local environmental temperature generated by physical therapies such as exercise, ultrasound, hyperthermia, and cold affect the expression and activity of YAP, thereby influencing the fate of cells.

Existing evidence predominantly concentrates on the role of YAP in physical therapy (Fig. 3). Notably, the Hippo/YAP signaling pathway encompasses crucial molecules, such as LatS1/2, NF2, and RASSF2. However, these molecules play a role in disease treatment but not undergone extensive examination. Hence, it is crucial to gain further understanding the involvement of these molecules in the transmission of mechanical stimuli in physical therapy. Furthermore, in addition to Hippo/YAP signaling, there are many other pathways can respond to mechanical stimulation or temperature change induced by physiotherapy, such as piezo1 and TRP channel proteins. Therefore, it is necessary to further investigate the mechanism by which physical therapy treat diseases by regulating other mechanical stimuli or temperature-sensitive proteins in future. Similarly, due to the specificity of physiotherapy, we only focused on the Hippo/YAP signaling pathway. In fact, there are many other molecules and signaling pathways that may be involved in the regulation of disease progression by physical therapy. Furthermore, clinically, the effects of physiotherapy are inconsistent at different stages of the disease. Nevertheless, there are no literature focused on the changes of YAP under mechanical stimuli in different disease stages at present. Besides, it is worth noting that except responding to mechanical stimuli, YAP is also an important transcription factor. In addition to physical therapy, materials such as hydrogel and drugs such as Dapagliflozin and Icariin have also been shown to affect the progression of diseases through regulating Hippo/YAP signaling pathway [134–137]. As mentioned above, temperature or mechanical sensitive materials can be used to treat diseases by regulating YAP. Therefore, it is significant to further explore the combination of physical therapy with drugs or special materials to treat diseases.

## Conclusion

In conclusion, Hippo/YAP signaling pathway represents a pivotal signaling pathway in the realm of physical therapy (Table 1). Particularly, physical therapy has gained extensive application in clinical practice for various diseases. Due to the ability to perceive mechanical stress within cellular microenvironments and convert it into chemical signals, Hippo/YAP signaling pathway provides theoretical basis to targeting disease treatment through physical therapy. However, the function of Hippo/YAP signaling pathway in different pathophysiological stages of diseases is still intricate and unclear. Therefore, additional research is essential to elucidate the mechanisms behind Hippo/YAP activation and regulation.

**Table 1:** The impact of physical therapy on disease via Hippo/YAP signaling pathway.

Physical therapy	Tissue (Cell) type	Signaling pathways	Effect	References
Exercise	human cardiac myocytes C57BL/6 mice	Hippo/YAP	promote stem cell migration and inhibit cell apoptosis	[61]
Aerobic exercise	SD rats	ELABELA-APJ-Akt/YAP	keep more cardiomyocytes alive and increase angiogenesis	[65]
FLIPUS	murine C2C12 cells	YAP	improve the cell proliferative potential	[77]
LIPUS	human umbilical vein endothelial cells	YAP/TAZ	initiate angiogenesis to accelerate fracture healing	[79]
LIPUS	C3H10T1/2 mesenchymal stem cell line	YAP	suppress differentiation into mature adipocytes	[81]
LIPUS	retinal ganglion cells mice ON crush model	YAP	protect retinal ganglion cells from loss and apoptosis	[82]
LIPUS	rat model of periodontitis, primary human PDLCs	YAP	inhibit hPDLC apoptosis and promote autophagic degradation	[84]
LIPUS	chondrocytes SD rants	YAP-RIPK1-NF-κB	rescue the damaged autophagy of chondrocytes	[85]
Periodic heat stress	ectomesenchymal stem cells	YAP/TG2	increase alkaline phosphatase activity and upregulate osteogenic-related proteins	[92]
Cold stress	293FT (or N2A) cells ICR pregnant mice RBM3 knockout mice	RBM3/YAP1	rescue the brain development defect	[93]
Cold stress	3T3-L1 cells and HEK293T cells CAG loxp-stop-loxp-Yap mice	miR-429-YAP	improve the impaired WAT browning in middle-aged mice	[95]
LTAP	human HaCaT cells, HDFs, NHEKs, NHEMs and murine B16F10 cells	YAP	regulate skin moisturizing and melanogenesis	[96]
Temperature-sensitive mechanically active hydrogel	rat model with full-thickness skin wounds	YAP and MEK	reduce inflammation	[102]
LIV	mesenchymal stem cells	YAP	restore basal nuclear YAP levels and increas the YAP nuclear shuttling	[116]
LIESWT	sciatic nerve injury model rat Schwann cells rat perineurial fibroblasts cells	YAP/TAZ	activate SCs and regenerate injured nerve axon	[123]
EA	SD rats	YAP	reduce apoptosis and neuroinflammation	[131]
EA	SD rats	YAP-OPA1	activate mitochondrial fusion function	[132]
EA	SD rats primary microglia	Hippo/YAP	regulate microglia polarization	[133]
